# PyDTS: A Python Toolkit for Deep Learning Time Series Modelling

**DOI:** 10.3390/e26040311

**Published:** 2024-03-31

**Authors:** Pascal A. Schirmer, Iosif Mporas

**Affiliations:** School of Physics, Engineering, and Computer Science, University of Hertfordshire, Hatfield AL10 9AB, UK; i.mporas@herts.ac.uk

**Keywords:** time series modelling, forecasting, nonlinear modelling, denoising, anomaly detection, degradation modelling, deep learning, machine learning

## Abstract

In this article, the topic of time series modelling is discussed. It highlights the criticality of analysing and forecasting time series data across various sectors, identifying five primary application areas: denoising, forecasting, nonlinear transient modelling, anomaly detection, and degradation modelling. It further outlines the mathematical frameworks employed in a time series modelling task, categorizing them into statistical, linear algebra, and machine- or deep-learning-based approaches, with each category serving distinct dimensions and complexities of time series problems. Additionally, the article reviews the extensive literature on time series modelling, covering statistical processes, state space representations, and machine and deep learning applications in various fields. The unique contribution of this work lies in its presentation of a Python-based toolkit for time series modelling (PyDTS) that integrates popular methodologies and offers practical examples and benchmarking across diverse datasets.

## 1. Introduction

Time series modelling has gained significant interest in the last decades due to the rise of machine learning and big data. It stands out as a crucial domain with diverse applications, ranging from financial forecasting to climate modelling [1,2]. The ability to analyse and forecast time series data has become increasingly important for timely informed decision making in various fields. Five different areas of applications can mainly be identified: first, denoising (or source separation), where the signal ground truth is isolated from a noisy observation, e.g., speech denoising [3] or separation of energy signals [4]; second, forecasting, where future signal values are predicted based on the signal’s history, e.g., grid load or weather forecasting [5]; third, nonlinear transient modelling, where nonlinear and possibly underdetermined problems are solved for time series inputs, e.g., transient thermal, structural, or fluid modelling [6]; fourth, anomaly detection, where outliers are identified in a large population of time series data, e.g., faulty samples in production sequences or failures under thermal/mechanical stress [7]; and fifth, degradation modelling, where a variable changes slowly over time, e.g., ageing of electric components and structures or expiration of food [8,9].

To model the above phenomena in time series signals, several mathematical approaches have been proposed in the literature. These approaches can be fundamentally split into three categories, namely, statistical, linear algebra, and machine- or deep-learning (ML, DL)-based ones. The dimensionality of the problem, i.e., the input and output dimension, as well as the problem evaluation over time, i.e., if the data have a constant mean value, highly determines which of the above techniques can be used to model the time series problem. For example, statistical models like autoregression or moving average processes are restricted to one-dimensional time series and have been applied to linear statistical problems and short-term ahead prediction [10]. Conversely, in the case of two or more variables, linear algebra models like state-space (SS) systems can be used to capture the input and output relation of multidimensional time series [11]. Most recently, machine and deep learning models have been used to capture complex multidimensional and possibly nonlinear relations between input and output samples of time series data [12], like long short-term memory (LSTM) [13], one-dimensional convolutional neural networks (CNNs) [14], or transformer models [15].

The topic of time series modelling has also been studied extensively in the literature. Modelling of statistical processes has been discussed in [16], with specific applications like wind speed modelling [17] or electricity or emission forecasting [18,19]. Similarly, state-space representations have been reviewed in [20]. In detail, state-space models have been proposed for thermal modelling in buildings [21] or battery electric vehicles [22], as well as in methodologies for solar irradiance forecasting in combination with exponential smoothing [23]. Moreover, numerous articles on machine and deep learning have been published covering the topics of feature extraction [24] and modelling approaches [25,26]. In specific, machine and deep learning approaches have been used for forecasting in applications like renewable energies [27], grid loads [28], and weather events [29]. Furthermore, deep learning models have been used for denoising in medical applications [30] and in renewable energy generation [31]. Similarly, nonlinear applications have been studied including structural dynamic problems [32], time delay approximations in optical systems [33], or transient thermal modelling [34]. Deep learning approaches have also been used in anomaly detection [35] and degradation modelling [36]. Most recently, also combinations of these approaches, e.g., deep state space models [37], or informed neural networks have been proposed [38]. Moreover, federated learning applications sharing one common model and approaches implemented on microprocessor hardware have been investigated [39].

Several different toolkits for time series modelling have been proposed previously, including Nixtla [40], AutoTS, Darts [41], and Sktime [42]. Each of these toolkits has a different purpose and different functionalities. While Nixtla and AutoTS only implement time series forecasting, Darts additionally implements anomaly detection, while Sktime implements forecasting, classification, regression, and data transformations. Likewise, PyDTS offers forecasting, classification, and regression functionalities, but additionally focuses on specific applications like denoising, nonlinear modelling, or degradation. The aim is to reduce the threshold of using deep-learning-based modelling as far as possible by offering a one-click functionality without needing to copy code, download, and preprocess data or plot results. The contributions of this article are as follows: First, the topic of time series modelling is reviewed. Second, a Python-based toolkit for time series modelling (PyDTS) with deep learning is presented, which incorporates the most used approaches and provides time series modelling examples for a wide range of datasets and benchmarking results. The results of these examples can be reproduced by calling one single function. Third, the article explains the effect of the free parameters, and the user can try these changes by simply changing one parameter without the need for changing the code while observing the changes based on a standard set of accuracy metrics and plots. Fourth, all results are evaluated on real-world datasets without the use of any synthetic or exemplary datasets. The toolkit is available on GitHub (https://github.com/pascme05/PyDTS, accessed on 27 February 2024).

The remainder of the article is structured as follows: In Section 2, a generalized architecture for time series modelling is described, also introducing the different applications of time series modelling. In Section 3, different modelling approaches are presented. An experimental setup and results for different datasets and applications are presented in Section 4. Finally, discussion and conclusions are provided in Section 5 and Section 6, respectively.

## 2. Time Series Modelling Architecture

**Figure 1:**
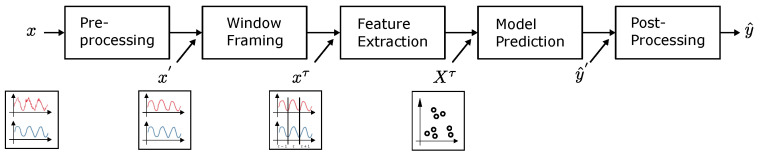
Generalized time series architecture.

**Figure 2:**
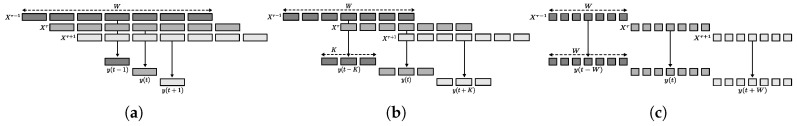
Relation between input and output dimensionality for frame-based time series modelling: (**a**) sequence-to-point, (**b**) sequence-to-subsequence, and (**c**) sequence-to-sequence.

As outlined in Section 2, time series modelling has several applications. In this section, a generalized modelling architecture is introduced, while specific approaches including their mathematical formulation are presented in Section 2.1–Section 2.5. Let us consider an input time series signal $x\in R^{T\times M}$ with *T* time samples of *M* input values each and a multivariate output signal $y\in R^{T\times N}$ with the same number of time samples and *N* output values; we can formulate the input–output relation as follows (1):

(1) $$y\left(t\right)=f_{\Theta }\left(x(t)\right),$$

where $f_{\Theta }\left(\cdot \right)$ is an arbitrary nonlinear function parametrized by a set of free parameters Θ. The goal of a time series modelling architecture is to model the input and output relation as in (2):

(2) $$\begin{array}{c} \begin{array}{c} \hat{y}\left(t\right)=g\left(x(t)\right)\\ s.t.\;\;min{∥y-\hat{y}∥}_{2} \end{array} \end{array}$$

where $g\left(\cdot \right)$ is an arbitrary regression or classification function aiming to approximate $f_{\Theta }\left(\cdot \right)$ and its free parameters, and $\hat{y}\in R^{T\times N}$ is the predicted output. The generalized architecture is illustrated in Figure 1:

As illustrated in Figure 1, the general architecture consists of five steps: first, preprocessing, e.g., resampling or filtering, of the raw feature input vector, *x* resulting into $x^{\prime }$; second, window framing $x^{\prime }$ into time frames $x^{\tau }\in R^{W\times M}$ with a window length *W*; third, feature extraction based on the time frame signals converting $x^{\tau }$ to a feature input vector $X^{\tau }\in R^{W\times F}$ with *F* input features; and finally, predicting and optionally postprocessing the model output $\hat{y}$. In specific, when predicting time series signals, the input and output relation can be modelled using three different approaches, which can be distinguished by their input and output dimensionality in the temporal domain. The three approaches are sequence-to-point modelling, sequence-to-subsequence modelling, and sequence-to-sequence modelling [43] and are conceptually illustrated in Figure 2.

**Figure 3:**
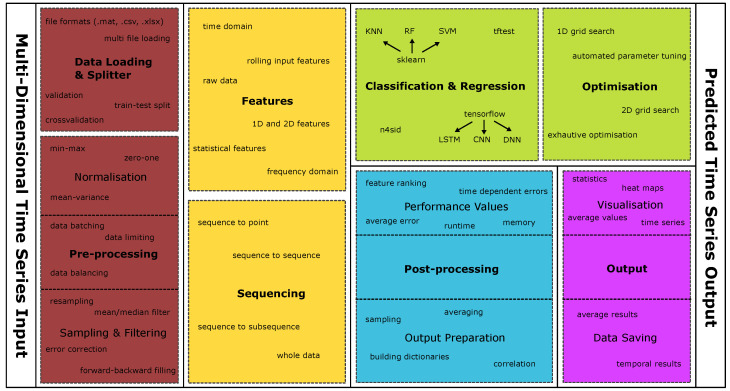
Overview of implemented modules and functionalities in the PyDTS toolkit. Inputs and preprocessing are indicated in red, features and data sequencing in yellow, modelling in green, postprocessing in blue, and visual elements and outputs in purple.

**Figure 4:**
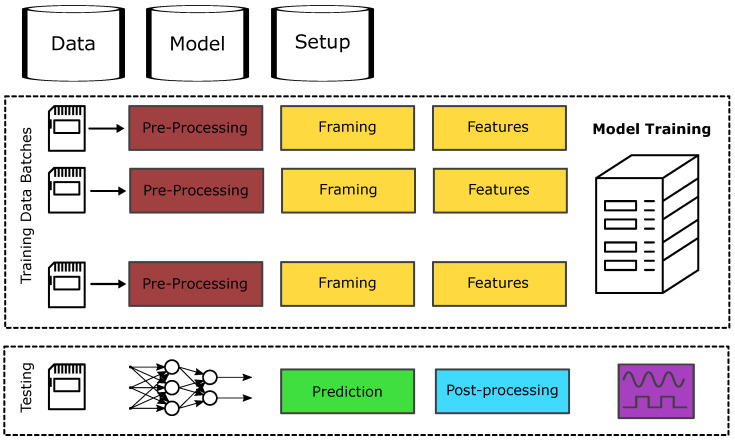
Internal data pipeline of PyDTS including training and testing modules and external data, model, and setup databases.

The PyDTS toolkit replicates the above structure, providing modules for preprocessing, framing, feature extraction, modelling approach, and postprocessing. The different modules offered by PyDTS and the flow diagram for the different operations are illustrated in Figure 3 and Figure 4.

In the following, the mathematical formulation of time series modelling with application in denoising, forecasting, nonlinear modelling, anomaly detection, and degradation modelling are provided.

### 2.1. Denoising

One of the most common time series prediction tasks is denoising, where the ground-truth data are retrieved based on a distorted observation. Without loss of generality, the problem can be formulated as in (3):

(3) $$y\left(t\right)=x\left(t\right)+\epsilon \left(t\right),$$

where y(t) is the output signal, x(t) is the input signal, and ϵ(t) is the noise. Here, we use as an example of denoising the energy disaggregation task, where appliance energy signatures (clean signal) are extracted from the aggregated data (noisy signal) [44]. Since multiple signals are extracted from a single observation, it is a single-channel blind source separation problem, i.e., a problem with very high signal-to-noise ratio. The problem can be mathematically formulated as in (4):

(4) $$y\left(t\right)=f\left(x_{m}\left(t\right),\epsilon \left(t\right)\right)=\sum_{m=1}^{M}x_{m}\left(t\right)+\epsilon \left(t\right),$$

where $y\left(t\right)$ is the aggregated signal, $x_{m}\left(t\right)$ is the *m*-th appliance signal, and ϵ(t) is additive noise from unknown devices, from electromagnetic interference on the transmission lines and from line coupling. The goal is to denoise the signal $y\left(t\right)$ by isolating the signature ${\hat{x}}_{m}\left(t\right)$ of each appliance.

### 2.2. Forecasting

Load forecasting is a task where future values, e.g., weather, energy consumption, or power draw, are predicted based on previous values of the same time series signal [45]. The aim is to model temporal information based on previous samples and accurately predict future values. Assuming linearity, the problem can be mathematically formulated as in (5):

(5) $$y\left(t\right)=\alpha y\left(t-1\right)+\beta x\left(t\right)+\epsilon \left(t\right),$$

where y(t) is the signal of interest, x(t) are signals with additional information and α,β are constant in the linear case, and ϵ(t) is stochastic noise. In this article, energy consumption prediction has been used as an example; i.e., future energy consumption values are predicted based on the consumption of previous days and additional information, e.g., weather or socioeconomic information [46].

### 2.3. Nonlinear Modelling

Nonlinear modelling is a task where the relation between input and output values is nonlinear. As an example application of nonlinear modelling, thermal modelling of power electronics and electric machinery is considered [47]. In this application, the fundamental heat conduction equation itself is linear, but nonlinearities are introduced through thermal coupling or losses, which are themselves a nonlinear function of temperature. Fundamentally, the temperature on a component can be modelled as in (6) and (7):

(6) $$\dot{q}\left(t\right)=R\left(\vartheta \right)\cdot I_{rms}^{2},$$

where $\dot{q}\left(t\right)$ is a time-dependent heat source that is generated by a current $I_{rms}$ flowing through a nonlinear temperature-dependent resistance R(ϑ). The temperature is then calculated using (7):

(7) $$\rho c_{p}\frac{\partial T}{\partial t}-\nabla \cdot \left(k\nabla T\right)=\dot{q}\left(t\right)\varphi \left(\vec{r}\right),$$

where ρ is the mass density, $c_{p}$ the specific heat capacity, and *k* the thermal conductivity. Furthermore, $\varphi (\vec{r})$ is a spatial function projecting the heat source $\dot{q}\left(t\right)$ on the respective volume.

### 2.4. Anomaly Detection

Anomaly detection describes the task of finding outliers within the data. Often, these data are highly unbalanced; i.e., there are much more positive than negative values or vice versa. The aim is to efficiently detect a small number of outliers within large amounts of time series data. The problem can be mathematically formulated as follows (8):

(8) $$\hat{y}\left(t\right)=\varphi \left(f_{\Theta }\left(x(t)\right)\right),$$

where $\hat{y}\left(t\right)\in 0,1$ is the anomaly detection status of the signal; i.e., if a sample at time *t* is normal or anomalous, x(t) are the input signals that provide indication for the status signal, $f\left(\cdot \right)$ is a function calculating the probability for a sample to be anomalous, and $\varphi \left(\cdot \right)$ is a threshold to convert the prediction into a binary variable. In this article, we used as an example model motor faults based on vibration data.

### 2.5. Degradation Modelling

Degradation modelling is a task where a relation between input parameters, time, and slow-varying output parameters exists. The aim is to describe the slow-varying degradation based on the initial state and the loads applied over time. The problem can be mathematically formulated as in (9):

(9) $$y\left(t\right)=y_{0}+\beta x\left(t\right)+\epsilon \left(t\right),$$

where y(t) is the degradation signal; x(t) are load signals stressing the component, e.g., temperature or mechanical stress; and ϵ(t) is stochastic noise. It must be noted that this problem depends on the initial state of $y_{0}$. In this article, the example case is to predict degradation data of lithium-ion batteries, i.e., the change of cell capacitance over time, using temperature, current, and voltage as input features.

## 3. Modelling Approaches

To implement the classification or regression function $f\left(\cdot \right)$ from (1), three approaches exist, namely, statistical, linear algebra, and machine or deep learning (ML, DL). In the following subsections, each of these three approaches is briefly explained.

### 3.1. Statistical Modelling

Assuming that the output function y(t) is a one-dimensional time series and only depends on previous values y(t−1) and stochastic white noise ϵ(t), then the relation between input and output can be expressed using statistical models based on autoregression and averaging (ARMA) [48], as described in (10):

(10) $$y\left(t\right)=c+\sum_{i=1}^{p}\phi_{i}y\left(t-1\right)+\sum_{j=1}^{q}\theta_{j}\epsilon \left(t-j\right)+\epsilon \left(t\right),$$

where *c* is a constant, $\phi_{i}$ is a weighting factor for the autoregression term, and $\theta_{j}$ is a weighting factor for the moving average.

### 3.2. Linear Algebra Modelling

If there are two processes, with one process being latent, thus describing a hidden time-varying structure, state-space representations have been used for the system identification of first-order systems with *M* inputs and *N* outputs [49]. The mathematical formulation for continuous parameter time-invariant coefficients is shown in (11):

(11a) $$\dot{s}\left(t\right)=As\left(t\right)+Bx\left(t\right)$$

(11b) $$y\left(t\right)=Cs\left(t\right)+Dx\left(t\right)$$

where $s\left(t\right)\in R^{L}$ and $\dot{s}\left(t\right)\in R^{L}$ are the internal system states and the derivatives with *L* being the number of states, $A\in R^{L\times L}$ is the system matrix, $B\in R^{L\times M}$ is the input matrix, $C\in R^{N\times L}$ is the output matrix, and $D\in R^{N\times M}$ is the feed-forward matrix. This model belongs to the category of white box modelling [50], where the states and the evolution of the states can be physically interpreted and, most importantly, also observed (12) and controlled (13) if the following restrictions are satisfied [49]:

(12) $$rank\left[B,\;AB,\;A^{2}B,\ldots ,A^{n-1}B\right]=L,$$

(13) $$rank\left[\begin{array}{c} \begin{array}{c} C\\ CA \end{array}\\ \begin{array}{c} \vdots \\ CA^{n-1} \end{array} \end{array}\right]=L,$$

### 3.3. Machine and Deep Learning

While the above techniques have limitations regarding the dimensionality of the input and output channels or the nonlinearity of the relation between input and output features, machine and deep learning models offer the highest flexibility in modelling an arbitrary function. In detail, the output of an artificial neural network with one hidden layer is shown in (14):

(14) $${\hat{y}}_{n}\left(t\right)=\varphi_{2}\left(\sum_{j=1}^{J}w_{nj}^{\left(2\right)}\varphi_{1}\left(\sum_{m=1}^{M}w_{jm}^{\left(1\right)}x_{m}\left(t\right)\right)\right),$$

where $\varphi_{1,2}\left(\cdot \right)$ and $w_{1,2}$ are the activation functions and the weights of the respective layer, and *J* is the number of nodes in the hidden layer. The weights can then be determined iteratively using backpropagation and a loss function, as shown in (15):

(15) $$E=\frac{1}{2n}\sum_{x}{∥y-\hat{y}∥}_{2}^{2},$$

### 3.4. Comparison

Each of the above modelling approaches has its advantages and disadvantages. A comparison list of relevant properties is shown in Table 1. Whenever, the respective property can be deducted directly from the model equation in Section 3.1–Section 3.3, e.g., the dimensionality of the input/output or the interpretability of the internal state. Table 1 lists the respective equation; otherwise, relevant literature is provided.

As can be seen in Table 1, machine and deep learning approaches suffer especially from larger computational complexity, memory requirements, and a lack of physical interpretation of the model parameters [50,51]. Statistical models present advantages, but at the same time, they are limited in 1D-only input and output dimensionality [48], as can be also seen from (10). This restriction makes statistical modelling approaches not feasible for most of the presented tasks in Section 2. In terms of transferability, deep learning approaches have very good transferability properties working as automated feature extraction engines [52]; however, they require extensive amounts of training data and have many hyperparameters to optimize [50,53]. Finally, as explained in Section 3.3, machine and deep learning models enable nonlinear modelling due to the nonlinear activation functions in (14). Because of the limitation of statistical and linear algebra models with respect to the input and output dimension in the following sections, the focus will be on machine and deep learning approaches.

## 4. Experimental Setup

The time series modelling architecture described in Section 2 was evaluated using the datasets, models, and experimental protocols presented below.

### 4.1. Datasets

The proposed time series prediction methods have been evaluated using publicly available datasets consisting of real-world data; i.e., no synthetic data have been used. In the following, each of the datasets is briefly explained. For disaggregation energy data (denoising), the AMPds2 dataset has been used, which includes 20 electrical appliances and the aggregated energy consumption of a Canadian household measured between 2012 and 2014 [54]. For energy consumption forecasting, the energy consumption of Tetouan, a city in the north of Morocco, has been used [55]. For nonlinear modelling, the motor temperature dataset in [47] has been used, which includes 185 h of measured temperatures of a state-of-the-art permanent magnet synchronous machine from a Tesla Model 3. To predict anomalies, motor vibration data have been used, which were previously classified into faulty and faultless motors [56]. To model degradation, the dataset from [57] was used, which includes lithium-ion battery cells measured over several cycles of charging and discharging under different conditions. The datasets, including their most important properties, are summarized in Table 2.

As can be seen in Table 2, the datasets cover a wide range of sampling frequencies, total number of samples, and input features, allowing for testing the PyDTS toolkit on different data inputs. Additionally, for the input features, the output that will be predicted is shown, as well as the max, mean, and standard deviation of the output. These values are included to provide a standard to the performance of the regression or classification models. For example, if the standard deviation of a dataset is close to zero, there are very few changes in the output signal; thus, a naive predictor would be sufficient to predict the outputs. Similarly, if the maximum predicted error of a model is equal to the maximum value of the output signal, while the average is close to zero, that indicates that the model is predicting well on average, but there are instances in which it fails to make an accurate prediction.

### 4.2. Preprocessing

**Figure 5:**
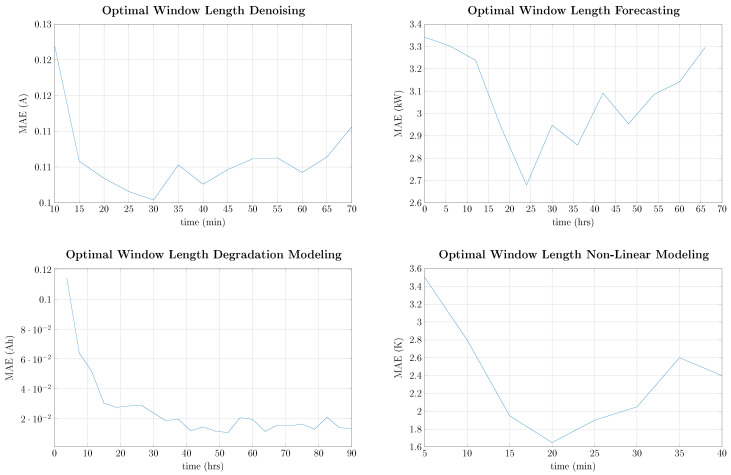
Grid search for the optimal number of input samples depending on the time series problem.

During preprocessing, the input data have been normalized using mean–std normalization for input features (16):

(16) $$x^{\prime }=\frac{x-\mu_{train}}{\sigma_{train}},$$

where $x^{\prime }$ is the input feature scaled by the mean ($\mu_{train}$) and standard deviation ($\sigma_{train}$) of the training data. Similarly, min–max normalization has been used for the output features (17):

(17) $$y^{\prime }=\frac{y-min(y_{train})}{max\left(y_{train}\right)-min\left(y_{train}\right)},$$

where $y^{\prime }$ is the output feature scaled by the minimum and maximum values of the training data. Furthermore, the optimal number of samples for the input window has been determined by grid search for each of the datasets tabulated in Table 1 with the exception of the anomaly detection as it is predefined in that dataset. The results are shown in Figure 5.

As can be seen in Figure 5, the optimal number of input samples strongly varies with the problem under investigation. In detail, when denoising electrical appliances signatures, the optimal input length is around 30 min, which is a typical operational duration for electrical appliances [58]. For the forecasting of electrical power consumption, the optimal input length was found to be around 24 h, which is typical due to working and living habits. It can also be observed that at around 12 h, 36 h, and 48 h, there are significant improvements. For modelling degradation data, no upper limit could be found since the degradation is a slow-varying property and it would be best to feed the complete degradation cycle at once, which is not possible due to the number of samples. The optimal input length for modelling the thermal behaviour of the electrical machine was found to be 20 min, which is in the order of the thermal time constant of the machine, and it is in line with [59]. Unless otherwise stated, the modelling approaches are based on sequence-to-point modelling using the optimized length of input samples from Figure 5, with one sample overlap between consecutive frames.

### 4.3. Model Structure and Parametrization

To implement the regression function $f\left(\cdot \right)$ for the approaches discussed in Section 2, different ML and DL approaches have been used. For ML approaches especially, random forest (RF) and K-nearest neighbours (KNN) have been evaluated, while for anomaly detection, also support vector machine (SVM) has been tested. The free parameters have been found using exhaustive automated parameter optimization on a bootstrap training dataset. The results are presented in Table 3.

**Figure 6:**
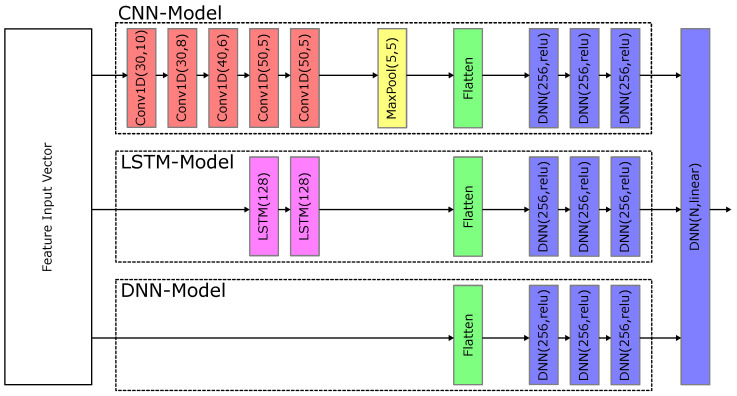
DL layer architectures for DNNs, LSTM, and CNN models. For CNNs, the notation of the convolutional layer is Conv1D(x,y) with x being the number of filters and y being the kernel size. For pooling layers MaxPool(x,y), x is the size and y the stride, while for LSTM and DNN layers, x denotes the number of neurons.

Similarly, for DL models, DNN, LSTM, and CNN architectures have been evaluated. The architectures are illustrated in Figure 6.

Unless otherwise stated, the above architectures have been used when being referred to CNN, LSTM, and DNN. For specific applications, the free parameters, i.e., the number of hidden layers, neurons, the kernel sizes, and the filters, have been optimized using the hyperband tuner from Keras. Additionally, the hyperparameters and solver parameters tabulated in Table 4 have been used.

## 5. Experimental Results

In this section, the experimental results are presented when using the data, the parametrizations, and models from Section 4. The results are evaluated in terms mean absolute error (MAE), root mean square error (RMSE), mean square error (MSE), and the normalized mean square error (NMSE):

(18) $$\mathrm{MAE}=\frac{1}{T}\sum_{t=1}^{T}\left|y\left(t\right)-\hat{y}\left(t\right)\right|,$$

(19) $$\mathrm{RMSE}=\sqrt{\mathrm{MSE}}=\sqrt{\frac{1}{T}\sum_{t=1}^{T}{\left(y\left(t\right)-\hat{y}\left(t\right)\right)}^{2}},$$

(20) $$\mathrm{NMSE}=1-\frac{\sum_{t=1}^{T}\left|y\left(t\right)-\hat{y}\left(t\right)\right|}{2\cdot \sum_{t=1}^{T}\left|y(t)\right|},$$

where $y\left(t\right)$ is the true signal, $\hat{y}\left(t\right)$ is the predicted value, and *T* is the total number of samples. Since not all modelling approaches are applicable for each of the scenarios, due to their limitations with respect to the input and output dimensionality, the following results are presented for machine and deep learning approaches. Each of these approaches can be reproduced with the PyDTS toolkit using the predefined configuration stored under the setup directory (https://github.com/pascme05/PyDTS/tree/main/setup/journal, accessed on 26 February 2024). Unless otherwise stated, the results were calculated using fivefold cross-validation using 10% of the training data for validation.

### 5.1. Denoising

For the denoising task, the energy of a Canadian household [54] has been disaggregated; i.e., the appliance-specific energy consumption has been extracted based on the observation of the total energy consumption of the household. Specifically, we focused on five different appliances: the dishwasher (DWE), the fridge (FRE), the heat pump (HPE), the wall oven (WOE), and the cloth dryer (CDE). For input features, active power (*P*), reactive power (*Q*), apparent power (*S*), and current (*I*) were used, while the output feature was the current for each device. The average results for all the five appliances and different machine and deep learning models are tabulated in Table 5.

As can be seen in Table 5, LSTM outperforms all other regression models for all accuracy metrics except for the maximum error. In this scenario, only 1D time series inputs were used to disaggregate the signals, and LSTM has shown outperforming results in application with 1D time series, including temporal information, i.e., where future samples depend on previous samples. Furthermore, the results for the best-performing model (LSTM) have been evaluated at the device level and are presented in Table 6.

As can be seen in Table 6, all appliances show low disaggregation errors, except the dishwasher, which shows poor performance that could be attributed to its lower activity, which is in line with other approaches reported on the same dataset [58]. Moreover, the results have been compared with the state-of-the-art approaches in the literature. The results are presented in Table 7.

**Figure 7:**
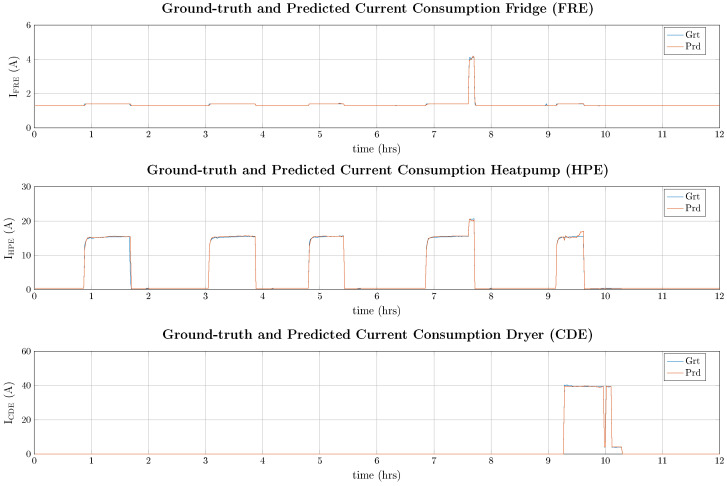
Predicted appliance current draw for 12 h for three different (FRE, HPE, and CDE) appliances from the AMPds2 dataset on 9 January 2013 at 12:00 p.m.

As can be seen in Table 7, the PyDTS toolkit reports results similar to the ones from previously reported approaches on the same dataset and is only outperformed by specifically optimized approaches for the energy disaggregation task. Moreover, a set of numerical predictions and ground-truth data is illustrated in Figure 7 for the best-performing LSTM model from PyDTS. In detail, a 12 h period with high appliance activity on 9 January 2013 at 12:00 p.m. was selected, where FRE, HPE, and CDE are active at the same time.

As can be seen in Figure 7, the LSTM model is able to extract all three appliance signatures from the aggregated data with high accuracy. There are only minor errors during the active periods where the current ripple is not precisely predicted.

### 5.2. Forecasting

For the forecasting task, the energy consumption of a city in Morocco [55] has been used. As input features, the previous power consumption values of the three-phase grid have been chosen. Additionally, these values have been extended by environmental features, namely, the ambient temperature, the wind speed, the relative humidity, and the solar irradiance. The output feature, which is predicted, is the power consumption on phase-leg L1. The results for an ahead forecast of 24 h are presented for different regression models in Table 8 using Seq2Point and in Table 9 using Seq2Seq approaches.

**Figure 8:**
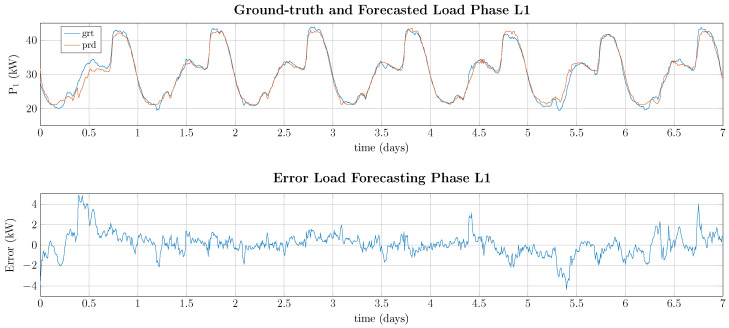
Forecasted power consumption and error for phase L1 for 1 week using RF as regression model.

As can be seen in Table 8 and Table 9, Seq2Seq approaches outperform Seq2Point approaches for all deep learning approaches with LSTM being able to capture the temporal relation reporting an average error equal to 2.36 kW. However, when considering Seq2Point approaches, RF shows improved performance reporting an average error of 1.60 kW but showing a significantly higher maximum error of 17.88 kW compared with the best-performing LSTM approach, which has a maximum error of 12.12 kW. The best performance is illustrated for 1 week in Figure 8.

As can be seen in Figure 8, the predicted power consumption is close to the actual value with errors between 1 and 5 kW. Interestingly, the errors at the beginning and ending of the week are higher than at the middle of the week, which is probably due to a higher fluctuation of power demand at these times.

### 5.3. Nonlinear Modelling

For the nonlinear modelling task, the temperature prediction of a permanent magnet synchronous machine [47] has been considered. In detail, four different temperature hot spots have been evaluated, namely, the stator winding, the stator tooth, the stator yoke, and the magnet temperature inside the rotor. As input features, the ambient and the coolant temperature, the stator current and voltages, and the mechanical torque as well as the rotational speed have been used. The output is the maximum stator winding ($\vartheta_{sw}$) and the rotor magnet ($\vartheta_{pm}$) temperature. The results in terms of MAE, RMSE, and MAX error are tabulated in Table 10 for stator and rotor temperatures, respectively.

As can be seen in Table 10, the rotor temperature shows worse performances across all models in terms of accuracy as its losses and thus temperatures are much more difficult to model based on the available inputs. Furthermore, deep learning models outperform machine learning models due to their ability to better capture the nonlinear relationship between the input feature vector and the temperature rise of the electric machine. To further compare the results, the experiments from [59] have been repeated using the same split for training, testing, and validation data. The results for the best-performing CNN model are tabulated in Table 11.

**Figure 9:**
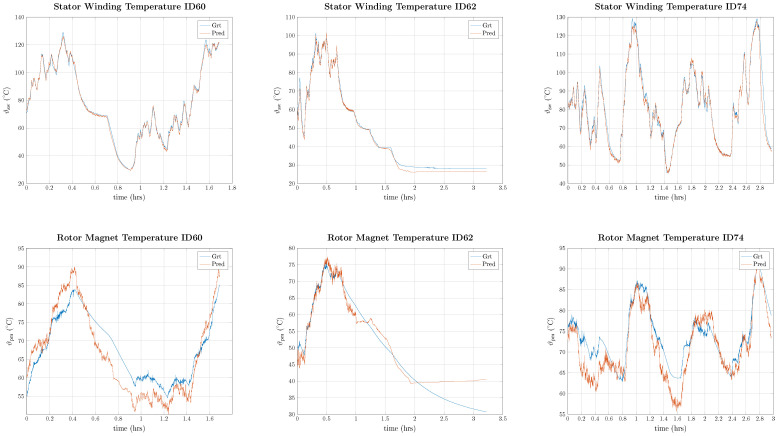
Predicted temperature for stator winding and rotor magnet for IDs 60, 62, and 72.

As can be seen in Table 11, the difficulty in estimating the temperatures in the different test IDs varies significantly, with the lowest errors being found in test ID 62 and the highest in test ID 72. On average, the results are better for the stator temperatures, which is in line with the input features being mostly stator quantities. In Figure 9, the temperature predictions for stator winding and magnet temperature are illustrated for all three testing IDs.

As can be seen in Figure 9, stator temperatures are much better predicted than rotor temperatures. Especially during heat-up and cool-down phases, the rotor temperature is not correctly predicted. This is probably due to the change in the heat transfer coefficient and the fact that the rotor is thermally isolated through the air gap; thus, the heat path is not based on heat conduction as in the stator, but a combination of heat convection and conduction. To compare the results with the previously published literature, a comparison of average errors was made in Table 12.

As can be seen in Table 12, the results obtained from the baseline CNN model implemented in PyDTS are comparable to the results obtained from other machine or deep learning architectures. Only physical informed approaches like thermal neural networks [59] perform significantly better.

### 5.4. Anomaly Detection

For the anomaly detection task, the vibration data of combustion engines, in normal and faulty states, have been used. As an input feature, the acceleration signal has been used, while the output is a binary variable indicating the healthy or faulty state of the motor [56]. Since, in this dataset, the training and test scenarios are presplit, the results will not be presented for fivefold cross-validation as in the previous experiments but using the predefined splitting of the data. In detail, the results were calculated three times, using raw input samples of the acceleration data, using statistical features of the acceleration data (mean, min, max, std, range, etc.) [44], and using frequency domain features (e.g., magnitudes of the Fourier transform signal or wavelets) [64,65]. The results in terms of accuracy (ACC) and F1-score (F1) are tabulated in Table 13 for different classification models.

**Figure 10:**
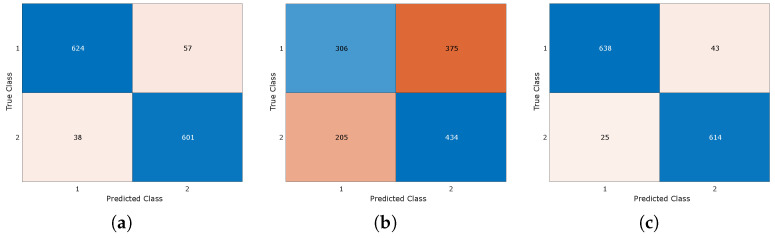
Confusion matrices for (**a**) raw, (**b**) statistical, and (**c**) frequency domain features for the CNN model.

As can be seen in Table 13, DL approaches clearly outperform ML-based approaches when using raw data operating as automated feature extraction engines. ML techniques show good results on frequency domain features as the relevant information is extracted when computing the Fourier coefficients. When using statistical features, none of the classification models can perform well, as the averaging effect in the time domain eliminates the vibration signatures discriminating healthy and faulty samples. To give more insights into the prediction accuracy, the confusion matrix of the best-performing CNN model is illustrated in Figure 10 for all three different feature setups.

### 5.5. Degradation Modelling

For the degradation modelling task, the ageing data of lithium-ion battery cells [57] have been used during charging and discharging. As input features, the cell current and voltage as well as the cell temperature have been used. The output is the degradation curve of the maximum remaining cell capacity for each charging and discharging cycle. The results for different regression models and accuracy metrics are tabulated in Table 14 for Seq2Point learning and in Table 15 for Seq2Seq learning. It must be noted that machine learning approaches are not able to perform Seq2Seq learning due to their restriction of the input dimensionality.

**Figure 11:**
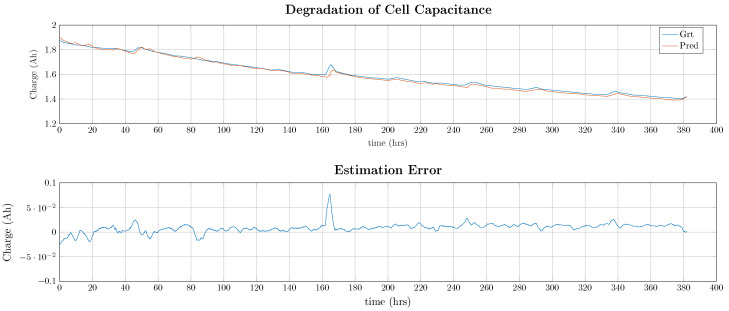
Ground-truth and predicted remaining cell charge and prediction error using the best-performing DNN model (for visibility, the predicted output has been filtered with a median filter of a length of 100 samples).

As can be seen in Table 14 and Table 15, deep learning approaches are significantly outperforming machine learning approaches due to their ability to model longer temporal characteristics. In detail, DNNs outperform all other models for all performance metrics except for the maximum error. The predicted degradation curve is illustrated in Figure 11.

As shown in Figure 11, the predicted output closely follows the measured degradation curve and is also capturing the frequent relaxation of the cell material, e.g., after 50 h. The maximum error is approximately 0.075 Ah being 12.3% of the remaining cell capacitance. On average, the model is underestimating the remaining capacity with around 0.01 Ah being 1.7% of the average cell capacitance.

## 6. Discussion

In this section, discussion on transferability is provided in Section 6.1, execution time and model size in Section 6.2, and model optimization and model order reduction in Section 6.3.

### 6.1. Transfer Learning

In transfer learning, the aim is to predict the output of new data based on a model that was pretrained on other data for a usually similar application. Two different approaches are investigated, namely, the intratransferability and the intertransferability. During intratransferability, the new data come from the same data domain, e.g., a different phase of the same electrical grid, while in intertransferability, the data only come from the same application domain, e.g., the same type of electrical appliance in a different consumer household. Both types of transferability will be considered in this subsection. The intratransferability setup is based on the electrical load forecasting of Section 5.2, predicting the load of phase 2 using a model trained on phase 1. The intertransferability setup is based on the disaggregation setup of Section 5.1 and [52], extracting the load signatures of a fridge, microwave, and dishwasher in a different household using the REDD dataset [66] (houses 1 and 2). The results for the intratransferability setup are tabulated in Table 16.

As can be seen in Table 16, the performance when predicting phase 2 based on a model of phase 1 leads to a decrease in all evaluated accuracy metrics and all regression models with a loss between 0.35% and 73.27%. However, due to the data coming from the same domain, the average accuracy is still relatively high between 87.44% and 93.28%. In detail, LSTM shows better performance capturing the temporal information of phase 1 and transferring it to phase 2, showing significantly lowest loss in accuracy by only 0.35–4.63%. The results for the intertransferability setup are tabulated in Table 17.

As can be seen in Table 17, the loss in performance is substantially increased compared with the intratransferability setup by 13.31–204.00%. This is due to the much more complex task of modelling similar devices in a completely different environment. Overall, CNN is achieving the best absolute performance for both the baseline and the transferability scenario.

### 6.2. Execution Time and Model Size

Model size and execution time determine the real-time capability and the utilization on hardware applications. Different models and application scenarios have been benchmarked on a personal computer using an AMD Ryzen 3700, an Nvidia RTX3070, and 32 GB of 3600 MHz DDR4 RAM. The model sizes after training are tabulated in Table 18.

From Table 18, it is observed that while the model size of CNN, LSTM, and DNN only depends on the size of the feature input vector, KNN stores all training samples to compute neighbouring distances and RF creates more trees, thus having significantly higher memory requirements for large datasets. Additionally, while the DNN and CNN models are sensitive to the window length of the input feature vector, the LSTM model has barely increased in model size due to its long short-term memory cells. The training and inference times are reported in Table 19.

As can be seen in Table 19, the training time per sample of deep learning approaches depends mainly on the convergence of the model. Conversely, the training time per sample for RF depends on the complexity and the number of different states that are extracted, while it is close to zero for KNN, which does not have any trainable parameters. Considering inference time, deep learning approaches are mostly dependent on the model size and the size of the input feature vector. Conversely, RF has very low inference time as it only performs comparison at the branches of the different decision trees, while KNN has large inference times because it compares every sample in the testing data with the training data.

### 6.3. Optimal Models and Model Order Reduction

**Figure 12:**
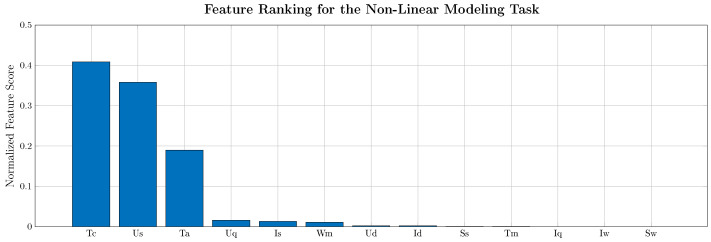
Feature ranking for the nonlinear modelling task for 13 features: coolant/ambient temperature ($T_{c}$, $T_{a}$), stator voltages ($U_{s}$, $U_{d}$, $U_{q}$), stator currents ($I_{s}$, $I_{d}$, $I_{q}$), torque ($T_{m}$), rotational speed ($\omega_{m}$), apparent power ($S_{s}$), and products or current/power and rotational speed ($I_{\omega }$, $S_{\omega }$).

To further improve the performance of a deep learning model in terms of model size and/or performance, the input feature vector and the model parameters can be optimized. To optimize the input feature vector, the importance of the input with respect to the output can be evaluated. Possible ranking algorithms include principal component analysis (PCA), correlation coefficients, or the ReliefF algorithm [67]. The feature ranking for the nonlinear modelling task is illustrated in Figure 12.

As can be seen in Figure 12, the stator and rotor temperature are dominated by the cooling temperature (heat conduction to the coolant), the ambient temperature (heat convection to the ambient), the stator voltage and stator current (ohmic and iron losses), and the rotational speed (coupling or stator and rotor temperature through airflow inside the machine). Furthermore, a Keras hyperparameter tuner can be used to optimize the parameters of the CNN model to account for the changed input feature dimensionality. The results of the reduced-order model using 6 input features instead of 13 are tabulated in Table 20.

As can be seen in Table 20, a reduced-order model reports even better performances for stator quantities, showing improvement by 34.1%. Conversely, the rotor performance decreased by 26.9%, which is probably due to the missing torque values and the complex power as these quantities are directly related to the rotor shaft.

## 7. Conclusions

A machine and deep learning Python toolkit for modelling time series data has been introduced. Five different scenarios, namely, denoising, forecasting, nonlinear modelling, anomaly detection, and degradation modelling, have been evaluated using real-word datasets and different machine and deep learning models. It was shown that the PyDTS toolkit and the models implemented in the toolkit can achieve performance close to the state of the art of the respective approach. Additionally, to benchmark the different approaches, the topics of transfer learning, hardware requirements, and model optimization have been discussed. The authors hope that the paper, accompanied by the PyDTS toolkit, will help new researchers entering the area of time series modelling and hopefully will create new ideas.

## Figures and Tables

**Table 1:** Comparison of relevant properties between different modelling approaches: (+): comparatively better, (o): neutral, and (-): comparatively worse.

Properties	Ref. and Eq.	Linear Algebra	Statistical Modelling	Machine Learning
Runtime	[51]	o	+	-
Memory	[51]	o	+	-
Interpretability	(12)–(14)	+	o	-
Dimensionality	(10), (11), (14)	o	-	+
Transferability	[52]	o	-	+
Nonlinear	(10), (11), (14)	o	-	+
Hyperparameters	(10), (11), (14)	o	+	-
Training data	[53]	+	o	-

**Table 2:** Short description of the datasets. The feature column includes the following abbreviations: active power (*P*), reactive power (*Q*), apparent power (*S*), current (*I*), voltage (*V*), temperature (*T*), relative humidity (RH), solar irradiance (IRR), wind speed (Ws), rotational speed (*n*), torque (*M*), and acceleration (*A*). Similarly, the outputs include the appliance current ($I_{app}$), the per-phase power ($P_{L_{x}}$), the stator winding and rotor magnet temperatures (ϑ), the motor state, and the remaining battery charge ($Q_{bat}$).

Name	Ref.	Scenario	Length	Sampling	Features	Output	Max	Mean	Std
AMPds2	[54]	Denoise	2 y	60 s	*P*, *Q*, *S*, *I*	$I_{app}$	105	0.8	10.9
Energy	[55]	Forecast	1 y	10 min	*P*, *T*, RH, WsIrr	$P_{L_{1},L_{2},L_{3}}$	52.2	23.7	12.2
Motor Temp.	[47]	Nonlinear	185 h	0.5 s	*V*, *I*, *T*, *M*, *n*	ϑ	141.4	57.5	22.7
Ford Motor	[56]	Anomaly	1.4 h	2 ms	$A_{x,y,z}$	*s*	1.0	0.49	0.50
Battery Health	[57]	Degradation	57 days	2.5 s	*V*, *I*, *T*	$Q_{bat}$	1.92	1.54	0.17

**Table 3:** Optimized model parameters for ML approaches including KNN, RF, and SVM.

Model	Parameter	Optimal	Range	Step
KNN	Neighbors	140	10–200	5
RF	Max. Depth	10	5-25	5
	Split	4	2–10	2
	#-Trees	128	2–256	$2^{n}$
SVM	Kernel	rbf	linear, rbf, poly	-
	C	100	1–200	20
	Gamma	0.1	0.001–1	$10^{n}$

**Table 4:** Hyper- and solver parameters for deep learning models including DNN, CNN, and LSTM.

Hyperparameters		Solver Parameters	
Batch	1000	optimizer	adam
Epochs	50–200	loss	mae
Patience	15	Learning rate	$1\times 10^{-3}$
Validation steps	50	Beta1	0.9
Shuffle	False	Beta2	0.999

**Table 5:** Average results (A) for the energy disaggregation task for fivefold cross-validation using different models and accuracy metrics. The best performances are indicated with bold notation.

Model	NMSE	RMSE	MSE	MAE	MAX
CNN	92.48	0.64	0.41	0.08	29.01
LSTM	**94.51**	**0.60**	**0.36**	**0.08**	30.54
DNN	94.39	0.66	0.44	0.08	31.85
RF	81.39	0.63	0.40	0.10	**28.60**
KNN	74.11	1.15	1.32	0.21	31.09

**Table 6:** Per-device results (A) for the energy disaggregation task for fivefold cross-validation using LSTM as regression model and different accuracy metrics.

Device	NMSE	RMSE	MSE	MAE	MAX
DWE	49.79	0.87	0.76	0.12	6.76
FRE	95.15	0.24	0.06	0.13	3.41
HPE	97.55	0.63	0.40	0.07	7.21
WOE	91.50	0.63	0.40	0.03	30.61
CDE	97.66	0.62	0.38	0.02	40.73
Avg	94.51	0.60	0.36	0.08	30.54

**Table 7:** Comparison with the literature for the energy disaggregation task.

Ref.	Year	Model	NMSE	RMSE	MAE
[60]	2016	HMM	94.1%	-	-
[61]	2019	CNN	93.9%	-	-
[62]	2020	CNN	94.7%	-	-
[58]	2021	CNN	95.8%	-	-
[43]	2022	CNN	94.7%	0.48	0.06
This Work	2023	LSTM	94.5%	0.60	0.08

**Table 8:** Forecasting errors (kW) using Seq2Point for a 24 h ahead prediction window with different models and accuracy metrics using fivefold cross-validation. The best performances are indicated with bold notation.

Model	NMSE	RMSE	MSE	MAE	MAX
CNN	95.72	3.62	13.10	2.77	18.49
LSTM	95.55	3.85	14.82	2.88	18.19
DNN	95.61	3.74	13.99	2.85	17.90
RF	**97.50**	**2.42**	**5.87**	**1.60**	**17.88**
KNN	93.98	4.96	24.60	3.88	18.63

**Table 9:** Forecasting errors (kW) using Seq2Seq for a 24 h ahead prediction window with different models and accuracy metrics using fivefold cross-validation. The best performances are indicated with bold notation.

Model	NMSE	RMSE	MSE	MAE	MAX
CNN	95.88	3.54	12.53	2.67	18.61
LSTM	**95.99**	**3.01**	**9.06**	**2.36**	**12.12**
DNN	95.66	3.71	13.76	2.81	17.26

**Table 10:** Temperature prediction results for 5-fold cross validation using different regression models and performance metrics. Due to memory restrictions the LSTM input was reduced to 500 samples. The best performances are indicated with bold notation.

Model	NMSE		RMSE		MSE		MAE		MAX	
	$\vartheta_{\mathrm{sw}}$	$\vartheta_{\mathrm{pm}}$	$\vartheta_{\mathrm{sw}}$	$\vartheta_{\mathrm{pm}}$	$\vartheta_{\mathrm{sw}}$	$\vartheta_{\mathrm{pm}}$	$\vartheta_{\mathrm{sw}}$	$\vartheta_{\mathrm{pm}}$	$\vartheta_{\mathrm{sw}}$	$\vartheta_{\mathrm{pm}}$
CNN	97.67	95.19	**4.54**	**7.59**	**20.61**	**57.61**	**3.06**	5.53	76.43	54.18
LSTM	96.71	93.23	6.39	10.6	40.83	112.4	4.28	7.85	77.15	60.05
DNN	**97.37**	**95.21**	5.32	7.81	28.30	61.00	3.43	5.59	76.52	59.20
RF	96.04	94.66	7.63	8.30	58.22	68.89	5.26	**4.43**	**73.73**	**47.87**
KNN	86.40	89.85	22.79	14.98	519.4	224.4	17.39	11.45	82.24	57.96

**Table 11:** Results for MSE (K²) and MAX (K) errors for different testing IDs, their respective time (hr), and temperature hot spots using a CNN regression model per hot spot.

ID	Time	Stator Winding		Stator Tooth		Stator Yoke		Magnet	
		MSE	MAX	MSE	MAX	MSE	MAX	MSE	MAX
60	1.7	2.41	5.03	1.68	4.28	1.16	3.14	22.62	9.90
62	3.3	2.75	6.23	1.25	3.78	1.22	3.96	17.49	9.74
74	3.0	3.33	6.18	2.42	5.43	1.80	5.00	14.47	10.81
Avg	8.0	2.90	6.23	1.78	5.43	1.42	5.00	17.45	10.81

**Table 12:** Comparison for temperature prediction using different models and number of input features.

Ref.	Year	Model	MSE	MAX	Features
[63]	2021	MLP	5.58	14.29	81
[63]	2021	OLS	4.47	9.85	81
[47]	2020	CNN	4.43	15.54	81
[59]	2023	TNN	2.87	6.02	5
This Work	2023	CNN	5.89	10.81	13

**Table 13:** Classification results in terms of ACC and F1 for anomaly detection using different classification models. The best performances are indicated with bold notation.

Model	Raw		Statistical		Frequency	
	ACC	F1	ACC	F1	ACC	F1
CNN	**92.35**	**92.34**	56.52	55.87	**94.85**	**94.85**
LSTM	51.06	50.52	55.30	54.90	51.59	35.12
DNN	80.15	80.15	56.52	56.13	94.77	94.77
RF	72.80	72.77	**59.09**	**59.10**	92.42	92.42
KNN	72.80	72.76	58.11	58.12	88.94	88.90
SVM	51.59	35.12	58.41	58.01	94.47	94.47

**Table 14:** Degradation errors for different regression models and performance metrics using Seq2Point learning. The best performances are indicated with bold notation.

Model	NMSE	RMSE	MSE	MAE	MAX
CNN	98.00	0.08	0.01	0.06	0.36
LSTM	97.85	0.08	0.01	0.07	0.39
DNN	**98.64**	**0.06**	**0.01**	**0.04**	0.49
RF	95.15	0.16	0.03	0.15	0.38
KNN	97.43	0.10	0.01	0.08	**0.35**

**Table 15:** Degradation errors for different regression models and performance metrics using Seq2Seq learning. The best performances are indicated with bold notation.

Model	NMSE	RMSE	MSE	MAE	MAX
CNN	**98.26**	**0.07**	**0.01**	**0.05**	**0.34**
LSTM	97.74	0.09	0.01	0.07	0.41
DNN	97.85	0.09	0.01	0.07	0.41

**Table 16:** Intratransferability scenario based on load forecasting between phases 1 (L1) and 2 (L2). The best performances are indicated with bold notation.

Model	L2 (Train L2)			L2 (Train L1)			Loss (%)		
	NMSE	RMSE	MAE	NMSE	RMSE	MAE	NMSE	RMSE	MAE
CNN	92.02	4.22	3.36	87.61	6.34	5.19	4.79	50.24	54.46
LSTM	93.21	3.58	2.81	92.88	3.70	2.94	**0.35**	**3.35**	**4.63**
DNN	92.81	3.86	3.03	87.44	6.40	5.25	5.79	65.80	73.27
RF	**96.02**	**2.35**	**1.71**	**93.28**	**3.44**	**2.78**	2.85	46.38	62.57
KNN	91.66	4.37	3.49	89.07	5.58	4.56	2.83	27.69	30.66

**Table 17:** Intertransferability scenario based on energy disaggregation between different consumer households (REDD-1,2). The best performances are indicated with bold notation.

Model	REDD2 (Train REDD2)			REDD2 (Train REDD1)			Loss (%)		
	NMSE	RMSE	MAE	NMSE	RMSE	MAE	NMSE	RMSE	MAE
CNN	**92.60**	39.44	**5.45**	**76.12**	70.83	**16.57**	16.48	79.59	204.0
LSTM	86.65	84.36	9.83	71.26	94.88	19.95	15.39	**12.47**	**102.9**
DNN	85.02	76.83	11.03	55.19	106.4	31.10	29.83	38.49	181.9
RF	89.19	41.38	7.96	75.88	**67.77**	16.74	**13.31**	63.77	110.3
KNN	92.48	**31.32**	5.54	70.09	79.57	20.76	22.39	154.1	274.7

**Table 18:** Model size of the trained model including all parameters for different scenarios.

Model	Denoise	Forecast	Nonlinear	Anomaly	Degradation
	30 × 4	144 × 8	1000 × 13	500 × 1	140 × 3
CNN	2.91 MB	6.37 MB	32.0 MB	9.49 MB	6.20 MB
LSTM	4.29 MB	4.30 MB	4.34 MB	4.26 MB	4.27 MB
DNN	1.92 MB	5.00 MB	40.6 MB	2.30 MB	2.81 MB
RF	37.7 MB	12.1 MB	58.4 MB	2.80 MB	9.16 MB
KNN	3.94 GB	0.33 GB	26.9 GB	7.05 MB	162.4 MB

**Table 19:** Training (T) and inference time (I) per sample (μs) for different models and scenarios.

Model	Denoise		Forecast		Non-Linear		Anomaly		Degradation	
	T	I	T	I	T	I	T	I	T	I
CNN	530	59	2570	120	2610	190	8650	478	1540	109
LSTM	540	87	6790	255	10,300	556	6540	893	2410	232
DNN	310	22	1500	33	3070	95	3760	76	1510	31
RF	9 × $10^{3}$	15	5710	5.5	20 × $10^{3}$	24	90	20	2170	3.1
KNN	0	6 × $10^{3}$	0	967	0	42 × $10^{3}$	0	97	0	854

**Table 20:** Temperature prediction results for stator winding and magnet temperature in terms of MSE (K²) for different testing IDs and models. Baseline scenarios are denoted with ‘Base’, while reduced-order configurations are denoted with ’MOR’.

ID	Time (h)	Stator Winding		Rotor Magnet	
		Base	MOR	Base	MOR
60	1.7	2.41	1.34	22.62	16.68
62	3.3	2.75	1.79	17.49	31.11
74	3.0	3.33	2.37	14.47	15.39
Avg	8.0	2.90	1.91	17.45	22.15

## Data Availability

The data on code are publicly available on GitHub at https://github.com/pascme05/PyDTS (accessed on 26 February 2024).
